# Retraction: Non-epigenetic function of HDAC8 in regulating breast cancer stem cells by maintaining Notch1 protein stability

**DOI:** 10.18632/oncotarget.27533

**Published:** 2020-03-24

**Authors:** Min-Wu Chao, Po-Chen Chu, Hsiao-Ching Chuang, Fang-Hsiu Shen, Chih-Chien Chou, En-Chi Hsu, Lauren E. Himmel, Han-Li Huang, Huang-Ju Tu, Samuel K. Kulp, Che-Ming Teng, Ching-Shih Chen

**Affiliations:** ^1^ Division of Medicinal Chemistry, College of Pharmacy and Comprehensive Cancer Center, Columbus, Ohio, USA; ^2^ Institute of Pharmacology, College of Medicine, National Taiwan University, Taipei, Taiwan; ^3^ Institute of Biological Chemistry, Academia Sinica, Taipei, Taiwan; ^4^ Department of Veterinary Biosciences, College of Veterinary Medicine, The Ohio State University, Columbus, Ohio, USA

**This article has been retracted:** The Program for Promotion of Research Integrity (PPRI), Academia Sinica, Taiwan, after twenty months of extensive and thorough investigation, has concluded that this paper should be RETRACTED, because of evidence showing that Dr. Ching-Shih Chen manipulated sixteen (16) images.

The investigation reports can be downloaded from this link (the two PDFs at the bottom of the page): https://ae.daais.sinica.edu.tw/site/datas/detail/2036/30/183/194/0

The Academia Sinica’s news release in English can be found here: https://www.sinica.edu.tw/en/news/6469

The Chinese version of the news release can be found here: https://www.sinica.edu.tw/ch/news/6469

Ohio State University officials reported that Dr. Ching-Shih Chen had manipulated images in at least 14 papers in different journals, eight of which required retraction.

As of today, 11 papers of Dr. Ching-Shih Chen were retracted from different journals.

Original article: Oncotarget. 2016; 7:1796–1807. 1796-1807. https://doi.org/10.18632/oncotarget.6427

